# New Drugs, Appliances, and Things Medical

**Published:** 1890-11-15

**Authors:** 


### NEW DRUGS, APPLIANCES, AND THINGS MEDICAL.

[All preparations, appliances, novelties, etc., of which a notice is desired, should be sent for The Editor, to care of The Manager, 140, Strand, London, W.C.]

### COCOA AND CHOCOLATE.

It is too late in the day to begin to praise cocoa and chocolate as if they were new and untried articles of food. Nevertheless, it is probable that very few people understand the real food value of cocoa, as compared with other dietetic substances. Nothing is more likely than that large numbers of persons believe cocoa and chocolate to be made from the dried and ground cocoa-nut. But, as a matter of fact, cocoa has nothing whatever to do with the ordinary cocoa-nut. Cocoa beans, from which cocoa and chocolate are made, not only do not grow on the cocoa-nut tree, but they grow on trees belonging to an entirely different family of plants. The cocoa-nut grows on a palm tree, which, of course, belongs to the palm order of plants. Cocoa beans are produced by trees of the genus Theobroma, which belong to the natural order Byttneriaceæ. Any student of elementary botany can tell us that there is as much difference between the cocoa-nut tree and the tree which produces cocoa beans as there is between a bracken and a gooseberry bush. The cocoa-nut is a very fine fruit, and yields an important commercial article known as cocoa-nut oil in large quantities. But the very name of the tree which yields the cocoa beans is striking evidence of the superior estimation in which it has been held from the earliest times until now. "Theobroma" means food for the gods, and "Theobroma Cacao" is the tree which yields cocoa and chocolate. "Cocoa-nut," or, as it was called until Dr. Johnson, in his sublime ignorance of natural science, altered the spelling, "Coco" is merely the Portuguese name for a "mask," and the Portuguese called the nut by this name, because its lower part looked, as they thought, so very like a mask.

"Theobroma Cacao" may very properly be called upon to account for its own name. On what ground does the cocoa bean claim to be a food for the gods? Well, here is a scientific justification. Let the reader notice the composition of roasted cocoa beans and compare it with that of beef steak:—

Cocoa-bean.

| | |
|---|---|
| (Hydro-carbon) Cocoa-butter ... ... ... | 51 |
| (Nitrogenous food) Gluten, albumen, &c. ... | 22 |
| (Carbo-hydrates) Starch, gum, &c. ... ... | 13 |
| Water ... ... ... ... ... ... ... | 5 |
| Mineral matter ... ... ... ... ... ... | 3½ |
| Cellulose ... ... ... ... ... ... ... | 3 |
| Theobromine ... ... ... ... ... ... | 2½ |
| | 100 |

Beef Steak.

| | |
|---|---|
| (Hydro-carbon) Fat ... ... ... ... ... | 4 |
| (Nitrogenous food) Gluten, albumen, &c. ... | 19 |
| Mineral matter ... ... ... ... ... ... | 5 |
| Water ... ... ... ... ... ... ... | 72 |
| | 100 |

Now, it does not require a person of very extraordinary scientific attainments to understand two points about the above tables. In the first place, the beef steak is nearly three-fourths water, and very little more than one-fourth solid; whilst the cocoa contains only one-twentieth part of water and nineteen-twentieths of solid. Moreover, the cocoa has a larger absolute quantity even of nitrogenous food than the meat, whilst in hydro-carbons it is more than twelve times richer. In addition, there is the active principle, theobromine, which gives its distinguishing characteristic to the cocoa food, and which, with the other constituents, justified Linnæus, as he thought, in calling cocoa "food for the gods." There are several excellent articles of this class in the market. Messrs. Cadbury and Co. recently sent us samples of their well-known cocoas and chocolates. The cocoa is pure, strong, and of a fine aromatic flavour. The Mexican chocolates are delicious and very digestible. There are two or three qualities of these chocolates, all good and well made; but the highest quality is particularly fine, and makes one wonder what name Linnæus would have given to Cadbury's delicious confection when he thought the crude bean worthy of so noble an appellation. Good cocoa is much more than a mere substitute for tea and coffee; it is both food and drink, and that of the most nourishing and easily digestible kind.

